# Heterotypic paracrine signaling drives fibroblast senescence and tumor progression of large cell carcinoma of the lung

**DOI:** 10.18632/oncotarget.10327

**Published:** 2016-06-30

**Authors:** Roberto Lugo, Marta Gabasa, Francesca Andriani, Marta Puig, Federica Facchinetti, Josep Ramírez, Abel Gómez-Caro, Ugo Pastorino, Gemma Fuster, Isaac Almendros, Pere Gascón, Albert Davalos, Noemí Reguart, Luca Roz, Jordi Alcaraz

**Affiliations:** ^1^ Unit of Biophysics and Bioengineering, Department of Biomedicine, School of Medicine, Universitat de Barcelona, Barcelona, Spain; ^2^ Tumor Genomics Unit, Department of Experimental Oncology and Molecular Medicine, Fondazione IRCCS Istituto Nazionale dei Tumori INT, Milano, Italy; ^3^ Medical Oncology Department, Hospital Clínic de Barcelona, Barcelona, Spain; ^4^ Anatomopathology Unit, Hospital Clínic de Barcelona, Barcelona, Spain; ^5^ Thoracic Surgery Unit, Hospital Clínic de Barcelona, Barcelona, Spain; ^6^ Thoracic Surgery Unit, Department of Surgery, Fondazione IRCCS Istituto Nazionale dei Tumori, Milano, Italy; ^7^ Institut d'Investigacions Biomèdiques August Pi i Sunyer, Barcelona, Spain; ^8^ Buck Institute for Age Research, Novato, CA, US; ^9^ CIBER de Enfermedades Respiratorias, Madrid, Spain

**Keywords:** lung cancer, large cell carcinoma, cancer associated fibroblast, senescence, invasion

## Abstract

Senescence in cancer cells acts as a tumor suppressor, whereas in fibroblasts enhances tumor growth. Senescence has been reported in tumor associated fibroblasts (TAFs) from a growing list of cancer subtypes. However, the presence of senescent TAFs in lung cancer remains undefined. We examined senescence in TAFs from primary lung cancer and paired control fibroblasts from unaffected tissue in three major histologic subtypes: adenocarcinoma (ADC), squamous cell carcinoma (SCC) and large cell carcinoma (LCC). Three independent senescence markers (senescence-associated beta-galactosidase, permanent growth arrest and spreading) were consistently observed in cultured LCC-TAFs only, revealing a selective premature senescence. Intriguingly, SCC-TAFs exhibited a poor growth response in the absence of senescence markers, indicating a dysfunctional phenotype rather than senescence. Co-culturing normal fibroblasts with LCC (but not ADC or SCC) cancer cells was sufficient to render fibroblasts senescent through oxidative stress, indicating that senescence in LCC-TAFs is driven by heterotypic signaling. In addition, senescent fibroblasts provided selective growth and invasive advantages to LCC cells in culture compared to normal fibroblasts. Likewise, senescent fibroblasts enhanced tumor growth and lung dissemination of tumor cells when co-injected with LCC cells in nude mice beyond the effects induced by control fibroblasts. These results define the subtype-specific aberrant phenotypes of lung TAFs, thereby challenging the common assumption that lung TAFs are a heterogeneous myofibroblast-like cell population regardless of their subtype. Importantly, because LCC often distinguishes itself in the clinic by its aggressive nature, we argue that senescent TAFs may contribute to the selective aggressive behavior of LCC tumors.

## INTRODUCTION

Non-small cell lung cancer (NSCLC) is the most frequent type of lung cancer and includes three major histologic subtypes: adenocarcinoma (ADC), squamous cell carcinoma (SCC) and large cell (undifferentiated) carcinoma (LCC), which account for ~40%, 30% and 5% of patients, respectively [1, 2]. Because all these NSCLC subtypes are epithelial in origin, most previous studies have focused on neoplastic epithelial cells. However, it is increasingly acknowledged that the aberrant tumor stroma that surrounds cancer cells supports cancer initiation, growth, stemness, invasion, metastasis and even resistance to therapies [3–5]. Accordingly, there is growing interest in understanding the molecular mechanisms underlying the tumor-promoting properties of stromal cells and in developing therapies targeting tumor-stroma interactions.

Tumor associated fibroblasts (TAFs) are frequently the most abundant cell type within the stromal microenvironment [3, 5]. Because the stroma in NSCLC and many other solid tumors is desmoplastic, the most common TAF phenotype is the myofibroblast-like, which is roughly characterized intracellularly by expression of alpha smooth muscle actin (α-SMA) and extracellularly by increased deposition of collagen and other fibrotic extracellular matrix (ECM) components [3, 6, 7]. Intriguingly, in addition to a myofibroblast-like phenotype, few recent studies have observed TAFs with a senescent phenotype in some types of breast, oral, liver and ovarian tumors [8–11].

A major hallmark of senescent cells is that they exhibit an irreversible growth arrest, yet they remain viable and metabolically active [12, 13]. In the context of cancer, a large body of work indicates that senescence acts as a physiologic protection against cancer cell expansion [5, 14]. In contrast, there is growing evidence that senescence in fibroblasts stimulates cancer cell proliferation in culture and tumor growth *in vivo* [8, 9, 13, 15–17]. Given their tumor-promoting effects, examining senescence in TAFs is drawing increasing attention. However, the presence and physiopathological relevance of senescent TAFs in NSCLC remains unknown.

To address this gap of knowledge, we examined common markers of senescence in primary TAFs from the 3 major NSCLC subtypes: ADC, SCC and LCC. Given the difficulties in gathering LCC-TAFs owing to the lower prevalence of LCC compared to the other subtypes, primary fibroblasts from 2 independent cell collections were used. We found an enrichment in myofibroblast-like TAFs regardless their histologic subtype, yet senescence was observed in LCC-TAFs only. Likewise, co-culture of normal lung fibroblasts with LCC (but not ADC or SCC) cells was sufficient to induce senescence, and this induction was mediated through oxidative stress. Of note, senescent fibroblasts provided growth and invasive advantages to LCC cells in culture and *in vivo* beyond those provided by control (non-senescent) fibroblasts, strongly supporting that they are essential contributors to the aggressive nature of LCC tumors.

## RESULTS

### Lung TAFs exhibit a myofibroblast-like phenotype regardless of their histological subtype, whereas senescence is restricted to LCC-TAFs

**Figure 1:**
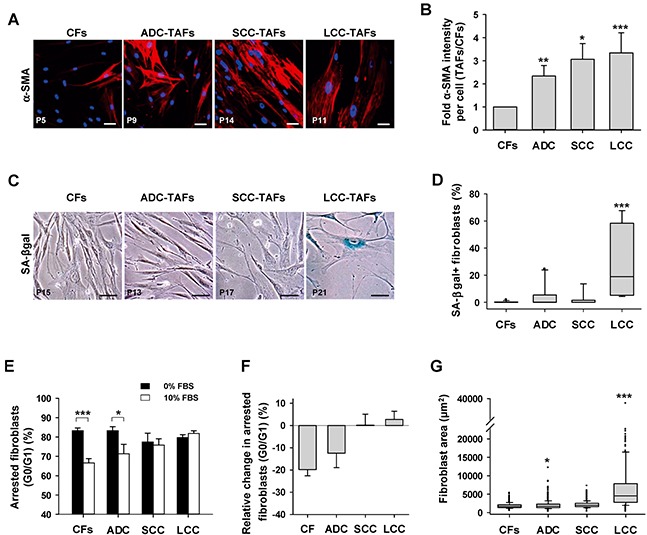
Analysis of myofibroblast and senescence markers in primary lung fibroblasts from major NSCLC subtypes (ADC, SCC and LCC) **A.** Representative fluorescence images of α-SMA stainings of cultured CFs and TAFs from a randomly selected patient of each histologic subtype. Patient number is indicated in the bottom-left of each image. Scale bar here and thereafter, 50 μm. **B.** Average fold α-SMA fluorescence intensity per cell of TAFs with respect to paired CFs for each subtype (6 ADC, 8 SCC, 3 LCC). Data shown as mean ± SE. **C.** Representative phase contrast images of SA-βgal stainings of cultured CFs and TAFs from a randomly selected patient of each histologic subtype. SA-βgal+ fibroblasts appear in blue. More images are shown in Supplementary Figure S1. **D.** Box-plot of the percentage of SA-βgal+ fibroblasts in CFs and TAFs for each subtype from two independent collections (10 ADC, 8 SCC, 4 LCC). **E.** Average percentage of growth arrested fibroblasts (G0/G1 of the cell cycle) in CFs and TAFs for each subtype (4 ADC, 4 SCC, 3 LCC) cultured with 0% and 10% FBS. **F.** Average relative change in arrested fibroblasts at 10% versus 0% FBS computed from the data in **G.** All pair-wise comparisons were performed with respect to CFs except in (E). Mann–Whitney rank sum test was used in (D). *, *P* < 0.05; **, *P* < 0.01; ***, *P* < 0.005 here and thereafter.

TAFs from the two major NSCLC subtypes (ADC, SCC) and other solid tumors exhibit an activated/myofibroblast-like phenotype in culture and *in vivo* [7, 18, 19]. Here we extended these observations by showing that LCC-TAFs are also activated and exhibit a statistically significant 3-fold increase in α-SMA expression with respect to paired CFs similar to that observed in ADC- and SCC-TAFs as shown by immunofluorescence analysis (Figure 1A, 1B). These results indicate that the myofibroblast-like phenotype is ubiquitous in NSCLC. In contrast, the percentage of fibroblasts positive for beta-galactosidase activity at pH 6, which is a widely used senescence marker [13], was much higher and statistically significant in TAFs compared to CFs from LCC patients only (Figure 1C, 1D and Supplementary Figure S1). Likewise, TAFs from LCC patients from 2 independent collections had percentages of senescence-associated beta-galactosidase activity positive (SA-βgal+) cells much higher than a ~3% consensus background [8, 20, 21]. Such high percentages of SA-βgal+ cells were found in LCC patients irrespective of their neuroendocrine status (Supplementary Table S1). In contrast, SA-βgal staining was largely absent (<< 3%) in CFs irrespective of their subtype, and reached percentages beyond background in only 20% and 10% of ADC- and SCC-TAFs, respectively (Figure 1C, 1D and Supplementary Table S1).

To further confirm the LCC-TAF-specific senescence enrichment, two additional senescence markers were examined: permanent growth arrest and enlarged spreading [12]. Cell cycle analysis by flow cytometry revealed that LCC-TAFs failed to exit growth arrest upon stimulation with 10% FBS compared to 0% FBS. Likewise, SCC-TAFs failed to enter into the cell cycle upon serum stimulation (Figure 1E–1F and Supplementary Figure S1). However, it is worth noting that we recently reported a marked mitogenic activity in SCC-TAFs in the absence of exogenous growth factors [18] that, in line with the lack of SA-βgal positivity, indicates that serum desensitization in SCC-TAFs is indicative of fibroblast dysfunction rather than senescence. In contrast, the percentage of growth arrested cells stimulated with 10% FBS drop by ~20% in CFs, and slightly less in ADC-TAFs (Figure 1F). In addition, LCC-TAFs exhibited a median spreading more than two-fold larger than all other groups (Figure 1G), in agreement with previous observations on senescent fibroblasts [13]. Therefore, all these data indicate that lung TAFs in culture are enriched in senescent cells selectively in LCC patients. Moreover, because TAFs from all subtypes were equally used at low passages, senescence in LCC-TAFs is likely to be indicative of premature aging rather than replicative exhaustion [12].

### Selective paracrine signaling with LCC cells is sufficient to “educate” normal lung fibroblasts to become senescent

**Figure 2:**
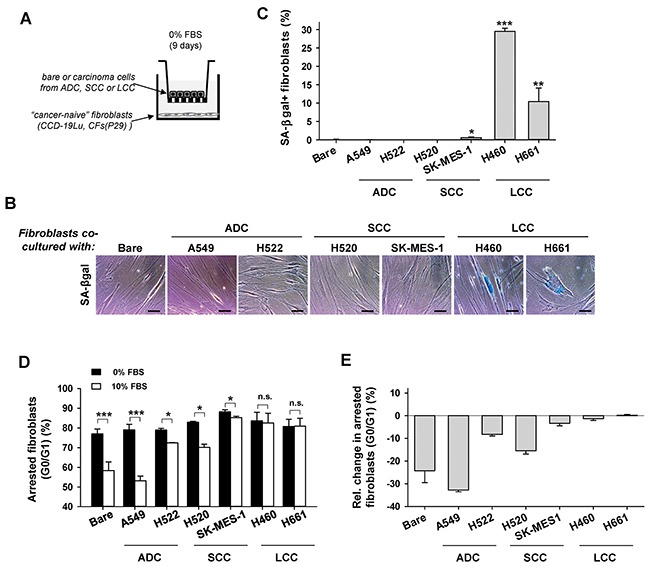
Analysis of senescence markers in normal (cancer-naive) lung fibroblasts co-cultured with lung cancer cell lines derived from ADC, SCC and LCC patients **A.** Outline of the Transwell-based co-cultures. **B.** Representative phase contrast images of SA-βgal stainings of CCD-19Lu fibroblasts co-cultured with a panel of lung cancer cell lines. More images are shown in Supplementary Figure S2. **C.** Average percentage of SA-βgal+ CCD-19Lu fibroblasts co-cultured with a panel of lung cancer cell lines. Bare Transwell inserts were used as negative control. All pair-wise comparisons were performed with respect to Bare. **D.** Average percentage of growth arrested CCD-19Lu fibroblasts co-cultured with a panel of lung cancer cell lines with 0% or 10% FBS. **E.** Average relative changes in growth arrested cells computed from data in (D) as in Figure 1G.

Tumor progression is increasingly regarded as the outcome of the aberrant co-evolution of both cancer and stromal cells through heterotypic signaling [3]. In line with this co-evolution framework, we examined whether paracrine interactions between LCC cells and cancer “naive” human lung fibroblasts (i.e. from non-malignant pulmonary tissue) was sufficient to induce senescence in the latter cells. For this purpose, normal human lung CCD-19Lu fibroblasts were co-cultured with three cancer cell lines from LCC patients (H460, H661 and H1299) in Transwells to enable indirect heterotypic paracrine signaling as outlined in Figure 2A. SA-βgal+ percentages larger than background were found in CCD-19Lu fibroblasts co-cultured with all LCC cell lines, with the highest percentage observed in H460 (Figure 2B–2C and Supplementary Figure S2). In contrast, percentages of SA-βgal+ fibroblasts comparable to the negative (Bare Transwell insert) control were found in fibroblasts co-cultured with a panel of ADC (A549, H522) and SCC (H520, SK-MES-1) cell lines (Figure 2C). In agreement with these observations, CCD-19Lu fibroblasts remained growth arrested upon stimulation with 10% FBS compared to 0% FBS after 9 days of co-culture with LCC cells, but not with either ADC, SCC cell lines or no cancer cells (Bare) (Figure 2D–2E). Interestingly, normal fibroblasts co-cultured with the SCC cell line SK-MES-1 exhibited a very modest decrease in growth arrested cells similar to that found in SCC-TAFs, revealing that SK-MES-1 is a suitable cell line to study abnormal SCC cell-stromal interactions. Moreover, positive markers of senescence (i.e. SA-βgal and permanent growth arrest) were observed in primary CFs from a randomly selected LCC patient (P29) co-cultured with LCC cells but not with ADC cells (Supplementary Figure S3). These results indicate that heterotypic paracrine signaling between LCC cells and normal lung fibroblasts is sufficient to induce selectively a senescent phenotype in the latter that is reminiscent to that found in LCC-TAFs.

### LCC cells induce fibroblast senescence through oxidative stress but not TGF-β signaling

**Figure 3:**
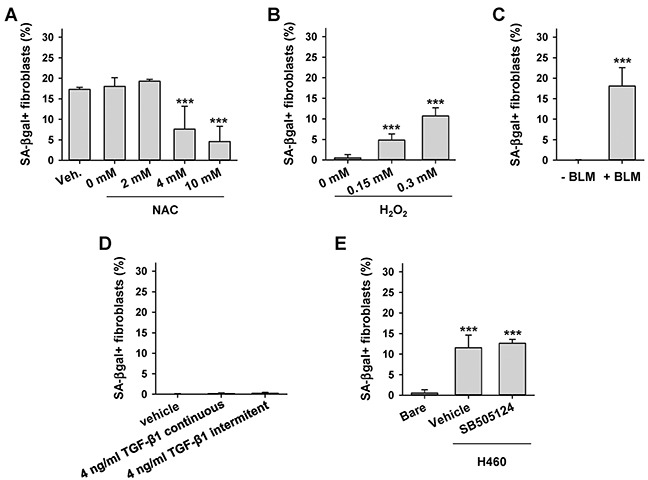
Effect of oxidative stress and exogenous TGF-β1 on fibroblast senescence induction by LCC cells **A.** Average percentage of SA-βgal+ CCD-19Lu fibroblasts co-cultured with H460 in the presence of increasing doses of the antioxidant NAC or vehicle. **B, C.** Average percentage of SA-βgal+ CCD-19Lu fibroblasts in response to direct or indirect oxidative stress elicited by (B) 2h treatment of H_2_O_2_ followed by 4 days of recovery or (C) 9 day treatment with bleomycin (BLM). **D.** Average percentage of SA-βgal+ CCD-19Lu fibroblasts daily treated with TGF-β1-continuously or intermitently for 4h/day as in [8]- for 2 weeks. **E.** Average percentage of SA-βgal+ CCD-19Lu fibroblasts co-cultured with H460 in the presence 5 μM of the TGF-β pathway inhibitor SB505124 for 9 days. All results correspond to two replicates from at least three independent experiments. All pair-wise comparisons were performed with respect to Bare or vehicle.

Premature fibroblast senescence has been associated with oxidative stress in chronic wounds and in TAFs from selected cancer subtypes [8, 9, 22]. In addition, the pro-senescent effects of oxidative stress in TAFs have been connected recently with long-term exposure to TGF-β1 in oral SCC [8]. To examine the involvement of these mechanisms in LCC-TAFs, we first co-cultured H460 LCC cells with CCD-19Lu fibroblasts in the presence of increasing doses of the antioxidant n-acetyl cysteine (NAC) and found a dose-dependent reduction of SA-βgal+ fibroblasts (Figure 3A). Alternatively, inducing oxidative stress in CCD-19Lu fibroblasts directly with H_2_O_2_ or indirectly with the chemoterapeutic DNA damaging drug bleomycin (BLM) [17, 23] was sufficient to induce a percentage of SA-βgal+ cells comparable to that induced upon co-culture with H460 cells (Figure 3B-3C). In contrast, daily exposure of CCD-19Lu fibroblasts to TGF-β1 -continuously or intermitently for 4h/day as in [8]- for 2 weeks failed to increase the percentage of SA-βgal+ cells beyond background (Figure 3D). Likewise, co-culturing H460 cells with CCD-19Lu fibroblasts in the presence of the TGF-β pathway inhibitor SB505124 failed to prevent senescence induction in fibroblasts co-cultured with H460 cells (Figure 3E). These results support that LCC cells induce senescence in fibroblasts through activation of oxidative stress. Moreover they reveal that oxidative stress is necessary and sufficient to induce premature senescence in lung fibroblasts through mechanisms other than activation of the TGF-β pathway.

### Conditioned medium from LCC-educated senescent fibroblasts provides growth and invasive advantages to LCC cells in culture

**Figure 4:**
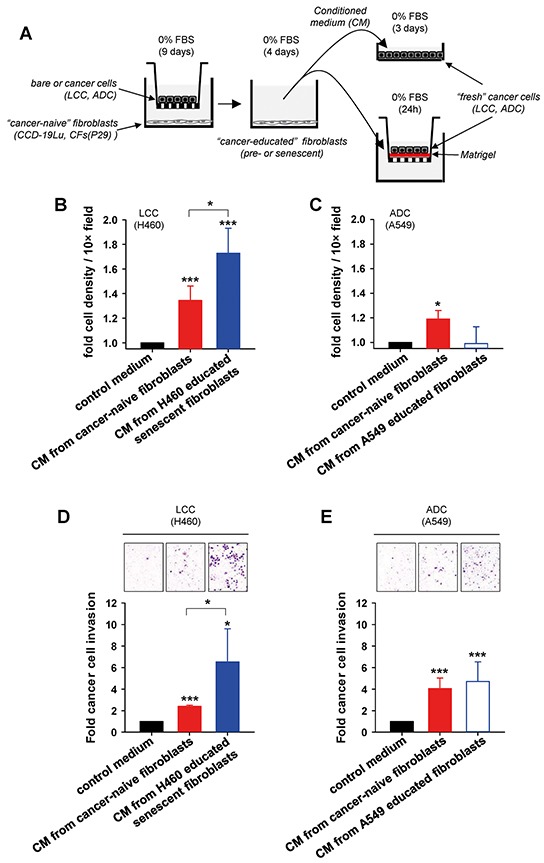
Assessment of the growth and invasive advantages provided by senescent fibroblasts to LCC cells in culture **A.** Outline of the experimental design used to assess growth (top right) and invasion (bottom right) in cancer cells in culture stimulated with conditioned medium (CM) from fibroblasts co-cultured as in Figure 2A. **B, C.** Average cell number/field of either (B) H460 LCC cells or (C) A549 ADC cells cultured with different types of conditioned medium as described in (A). Results were normalized to control medium, and are shown as mean ± SE. **D, E.** Average invasion of either (D) H460 cells or (E) A549 cells induced by different types of conditioned medium as in (B,C) assessed by area quantification of crystal violet positive invasive cells (top images). Results were normalized to control medium. All results correspond to at least two replicates from three independent experiments. All pair-wise comparisons were performed with respect to control medium unless otherwise indicated.

LCC is among the most aggressive NSCLC subtypes, for it tends to grow and spread more quickly than other subtypes [1, 24]. Given the previously reported protumorigenic effects of senescent fibroblasts in other cancer types [8, 9, 13, 15–17], and the aggressive nature of LCC, it is conceivable that LCC-educated senescent fibroblasts may provide a selective growth and/or invasive advantage to LCC cells. To examine this possibility, we first analyzed the growth of H460 LCC cells as outlined in Figure 4A. After 3 days of culture, conditioned medium (CM) from cancer-naive fibroblasts increased the population of H460 cells by ~35% with respect to H460 cells cultured in their regular serum-free medium (used as control medium). Remarkably, CM from H460-educated senescent fibroblasts increased the population of H460 cells by ~70%, i.e. twice as much as CM from cancer-naive fibroblasts (Figure 4B). Qualitatively similar results were found in two independent LCC cell lines (H1299 and H661) cultured in the same 3 conditions and in H460 LCC cells cultured with CM from cancer-naive or H460-educated senescent CFs from a randomly selected patient (P29) (Supplementary Figure S4). In contrast, H460 LCC cells did not increase their number when cultured with CM from CCD-19 fibroblasts co-cultured with A549 ADC cells (Supplementary Figure S4). Likewise, A549 ADC cells did not exhibit any significant growth advantage when cultured with CM from A549-educated fibroblasts compared to control medium, and only grew modestly with CM from cancer-naive fibroblasts (Figure 4C), in agreement with independent studies reported elsewhere [25].

Next, we examined the invasive capacity of cancer cells through reconstituted basement membrane (Matrigel) using a Transwell-based assay as outlined in Figure 4A. In agreement with the latter growth data, CM from H460 educated senescent fibroblasts markedly enhanced the invasion of H460 LCC cells compared to serum-free control medium, and well beyond the effects induced by cancer-naive fibroblasts (Figure 4D). Comparable results were obtained in two LCC cell lines (H1299 and H661) (Supplementary Figure S4). In contrast, CM from fibroblasts provided an invasive advantage to A549 cells compared to control medium irrespective of the senescence status of the fibroblasts (Figure 4E). These findings reveal that soluble factors secreted by LCC-educated senescent fibroblasts are sufficient to elicit selective growth and invasive advantages to LCC cells beyond those provided by (non-senescent, non-activated) cancer-naive fibroblasts.

### LCC-educated senescent fibroblasts provide a growth and dissemination advantage to LCC cells *in vivo*

**Figure 5:**
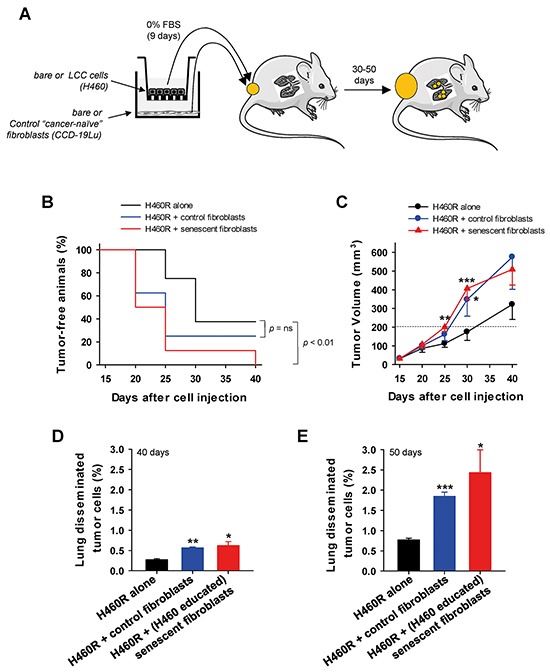
Assessment of the growth and invasive advantages provided by senescent fibroblasts to LCC cells *in vivo* **A.** Outline of the experimental design used to assess tumor growth and lung cancer cell dissemination of H460R LCC cells subcutaneously co-injected alone, with cancer-naive fibroblasts, or with H460-educated senescent CCD-19Lu fibroblasts into immunedeficient mice. **B.** Percentage of tumor-free flanks monitored over 40 days, i.e. tumors lower than 200 mm^3^. **C.** Tumor volume monitored over 40 days (n = 8 tumors per condition). Data are mean ± SE. (D,E) Percentage of lung disseminated tumor cells at **D.** 30 and **E.** 50 days after injection. All pair-wise comparisons were performed with respect to H460R injected alone. Statistical significance in (B) was assessed by log-rank test.

The growth and invasive advantages provided by senescent fibroblasts to LCC cells in culture prompted us to examine whether such advantages could be observed *in vivo* as outlined in Figure 5A. We first confirmed that H460 cells in co-cultures can induce senescence of primary mouse skin fibroblasts derived from the same strain used in the *in vivo* assays (Supplementary Figure S6 and Supplementary Material). H460 LCC cells exhibited a similarly marked tumor take and growth when co-injected subcutaneously into immunodeficient mice alone or with fibroblasts regardless the senescence status of the latter (Supplementary Figure S5). These observations are consistent with a recent study from some of us showing that the rapid growth and aggressive nature of H460 cells may override additional tumor-promoting signals from fibroblasts [26]. Alternatively, we used H460 LCC cells transfected with a miR-200c mimic (referred to as H460R), which are more responsive to microenvironmental signals *in vivo* than parental H460 cells [26], yet they retain both the ability to induce senescence when co-cultured with either human lung fibroblasts or primary mouse skin fibroblasts, and to provide growth and invasive advantages to LCC cells in culture (Supplementary Figure S6). All mice subcutaneously co-injected with H460R cells and senescent fibroblasts developed tumors after 40 days of injection, and exhibited a statistically significant different time evolution with respect to H460R cells injected alone. In contrast, tumor take evolution was not statistically different between H460R cells co-injected alone or with cancer-naive fibroblasts, and increased similarly up to 60-70% during the 40 day observation period in both conditions (Figure 5B). Likewise, H460R cells co-injected with senescent fibroblasts elicited larger tumors than H460R cells injected either alone or with control fibroblasts as early as 25 days after injection. Five days later, H460R cells co-injected with control fibroblasts elicited also larger tumors than H460R alone, altough the statistical significance of such difference was lower (*P* < 0.05) than that attained when co-injecting with senescent fibroblasts (*P* < 0.005) (Figure 5C). The differences in tumor size between H460R injected alone or with control or senescent fibroblasts were reduced at the end of the observation period, which can be easily explained by the rapid growth of cancer cells compared to fibroblasts, and by the likely involvement of host stromal cells at longer times in all conditions [27].

To further assess the differential aggressiveness of LCC cells co-injected with senescent fibroblasts *in vivo*, we analyzed lung dissemination of tumor cells using a FACS-based approach recently reported by some of us [26]. As early as 20 days after the tumor latency seen in Figure 5B-5C (day 40 after injection), the percentage of lung disseminated tumor cells was already more than 2-fold higher in mice bearing H460R co-injected with cancer-naive fibroblasts than in H460R injected alone, and was even ~10% higher when co-injected with senescent fibroblasts compared to cancer-naive fibroblasts (Figure 5D). Remarkably, the latter differential induction of lung disseminated tumor cells elicited by cancer-naive fibroblasts was maintained and even increased up to 30% at 30 days after tumor latency (i.e. 50 days after injection) in a different group of mice, and collectively all percentages of disseminated tumor cells rose ~3-fold (Figure 5E). Thus, even though cancer-naive fibroblasts co-injected with H460R cells stimulated markedly both tumor growth and lung dissemination compared to H460R injected alone, it is remarkable that such stimulation was further increased when co-injecting with senescent fibroblasts. These results strongly support that senescent fibroblasts elicit a more aggressive phenotype to H460R cells *in vivo* than normal fibroblasts, in agreement with our observations in culture.

## DISCUSSION

Given the ubiquitous myofibroblast-like phenotype in the stroma of solid tumors, it is unsurprising the common assumption that lung TAFs are a heterogeneous population of myofibroblast-like cells regardless their histologic subtype [28, 29]. However, we recently challenged this assumption by reporting strikingly distinct responses to extracellular matrix stiffening and growth factor stimulation between ADC- and SCC-TAFs, which were associated with subtype-specific β1 integrin expression and FAK activity [18]. In the present study we expanded these observations further by reporting for the first time two new subtype-specific aberrant phenotypes in lung TAFs: (i) poor growth response in the absence of senescence markers in SCC-TAFs, and (ii) premature cellular aging or senescence in LCC-TAFs. In addition, we identified SK-MES-1 and H460 as suitable lung cancer cell lines to reprogram normal fibroblasts towards the acquisiton of the latter two aberrant phenotypes observed in SCC- and LCC-TAFs, respectively. Interestingly, similar aberrant phenotypes have been reported in fibroblasts from chronic (non-healing) wounds, and pointed as essential contributors to the wound chronicity [22, 30, 31]. Thus, our findings begin to define the subtype-specific stromal similarities between lung tumors and chronic wounds, and reveal convenient preclinical culture models to study subtype-specific cancer-stroma interactions in the context of SCC and LCC.

Epidemiologically, increased cellular senescence has been linked with aging [13]. However, even though virtually all our patients were > 55 year old, aging alone could not explain the selective appearance of senescent TAFs in LCC, for CFs from the same patients were not senescent. On the other hand, premature senescence in lung fibroblasts has been previously associated with cigarette smoke [21] and emphysema [32]. However, our selected patients were all current smokers and exhibited different degrees of lung emphysema, yet CFs and most TAFs were negative for senescence markers in ADC and SCC patients, indicating that the premature senescence of LCC-TAFs was attributed to neither cigarette smoke nor emphysema, but rather to LCC-specific processes.

At the mechanistic level, a large body of work indicates that senescence can be induced by either telomere-dependent or independent processes. The lack of senescence markers in CFs from LCC patients and the strong inhibition of fibroblast senescence when co-cultured with LCC cells in the presence of increasing doses of the antioxidant NAC strongly support that senescence in LCC-TAFs was due to a telomere-independent process mediated by oxidative stress from LCC cells. In support of this interpretation, inducing oxidative stress in normal lung fibroblasts directly by H_2_O_2_ or indirectly by the DNA damage drug bleomycin was sufficient to render them senescent, in agreement with previous studies with fibroblasts from other tissue types [17]. Likewise, oxidative stress elicited by secreted factors from oral SCC cells was found necessary for senescence induction in normal oral fibroblasts [8]. However, the latter study reported that fibroblast senescence was selectively induced by genetically unstable (p53 deficient) oral SCC cells but not by wild-type p53 cells, and was mediated by sustained TGF-β exposure. In contrast, H460 LCC cells induced the largest percentage of senescent lung fibroblasts in co-culture experiments yet they have wild-type p53. Conversely, most of the ADC and SCC cell lines used in our study have mutant p53 yet they failed to induce senescence in fibroblasts (Figure 2C and Supplementary Table S2). Likewise, long-term exposure to exogenous TGF-β1 alone did not induce premature fibroblast senescence, and inhibiting the TGF-β pathway in co-cultures of LCC cells with fibroblasts failed to prevent fibroblast senescence. These results illustrate the ability of LCC cells to selectively “educate” fibroblasts to become senescent irrespective of their p53 status, and reveal that such “education” is mediated, at least in part, through oxidative stress-dependent and TGF-β pathway-independent signaling. Elucidating the molecular mechanism by which the crosstalk between LCC cancer cells and fibroblasts induce oxidative stress-dependent senescence in fibroblasts was beyond the scope of this study. However, potential mechanisms include either direct or indirect induction of oxidative stress by extrusion of reactive oxygen species (ROS) or secretion of non-ROS signaling factors by cancer cells, respectively [8, 9].

Even though normal (pre-senescent) fibroblasts provided notable growth and invasive advantages to LCC cells in culture, such advantages were further increased by cancer-educated senescent fibroblasts in three independent LCC cell models. Likewise, the largest tumor take, tumor growth and lung dissemination of tumor cells *in vivo* were consistently observed when LCC cells were co-injected with senescent fibroblasts (Figure 4). In agreement with our observations, increased tumor sizes have been reported in xenografts of cancer cells co-injected with senescent compared to pre-senescent fibroblasts in other cancer cell models [15, 27, 33–35]. Likewise, breast cancer cells co-injected with fibroblasts exhibiting an autophagic/senescent phenotype into the tail veins of nude mice elicited increased lung colonization compared to that elicited by control fibroblasts [9]. In contrast, to our knowledge we are the first to report enhanced tumor cell dissemination to the lungs from primary tumor xenografts using cancer cells co-injected with senescent fibroblasts. These observations strongly support that premature senescence in TAFs may potentiate the aggressive nature of a tumor, at least in terms of growth and dissemination, which is in marked contrast with the well-known tumor suppressive role of senescence in cancer cells [5, 14].

LCC tumors, particularly those positive for neuroendocrine markers, often distinguish themselves in the clinic by their aggressive behavior. However, the mechanisms underlying the aggressive features of LCC tumors remain fairly unknown. In this context, it is worth noting that protein biomarkers for senescence and oxidative stress in the tumor stroma have been associated with poor clinical outcome and drug-resistance in human breast cancer patients [9, 36]. On the other hand, we found a selective premature senescence in LCC-TAFs in culture, and that senescent fibroblasts provide growth and invasive advantages to LCC cells in culture and *in vivo* beyond those provided by non-senescent fibroblasts. Based on these lines of evidence, it is tempting to speculate that senescent TAFs may support, at least in part, the aggressive nature of LCC tumors.

Defining how senescent fibroblasts contribute to disease progression is drawing increased attention not only in cancer, but also in chronic wounds and other diseases [13, 22]. Elucidating the specific tumor promoting mechanisms of senescent TAFs in LCC was beyond the scope of our work. However, previous studies have shown that senescent fibroblasts exhibit a secretory phenotype or SASP enriched in factors involved in numerous processes including epithelial growth, ECM degradation, and inflammation [13, 37]. Our *in vivo* data are consistent with the first two processes. Likewise, additional support for an important role of growth factors and ECM degrading enzymes secreted by senescent fibroblasts in tumor progression has been reported elsewhere [17, 27, 34]. Thus, future studies are warranted to examine the biological causes and clinical implications of the selective premature senescence of LCC-TAFs revealed in this study.

## MATERIALS AND METHODS

### Lung tissue samples and primary human lung fibroblasts

Primary fibroblasts were obtained from two independent collections. In the first collection, tissue samples were gathered from 19 resected lung specimens from early stage NSCLC patients at the *Hospital Clinic de Barcelona* (HCB, Spain) (8 ADC, 8 SCC, 3 LCC). The study was approved by the Ethics Committees of the HCB and the *Universitat de Barcelona*, and all patients gave their informed consent. Selected patients were male, chemo-naïve, Caucasian, ≥ 55 years old, and current smokers. A fraction of each tissue sample was paraffin-embedded, and the remaining was used to isolate fibroblasts by outgrowth of tissue explants [38]. Samples from tumor and paired tumor-free lung parenchyma were used to obtain tumor associated fibroblasts (TAFs) and control fibroblasts (CFs), respectively. In the second collection, tumor samples were gathered from 5 resected NSCLC patients at the *Fondazione IRCCS Istituto Nazionale dei Tumori* in *Milano* (INT, Italy) (2 ADC, 2 SCC, 1 LCC), with the approval of the Ethics Committee of the *Fondazione IRCCS INT* and with the informed patient consent. The patient selection criteria were similar to those used in the first collection, and fibroblasts were isolated by collagenase digestion [39]. The mesenchymal nature of the fibroblasts was confirmed by their positive and negative immunofluorescence staining with mesenchymal and epithelial/hematopoietic antibodies, respectively (more details in Supplementary Material). A summary of clinical patient data is shown in Supplementary Table S1.

### Cell culture

The human fibroblast cell line CCD-19Lu obtained from normal pulmonary tissue (ATCC) and primary fibroblasts were maintained in DMEM-based fibroblast culture medium described elsewhere [18, 39]. Fibroblasts were used up to either passage 10 (CCD-19Lu) or 6 (primary) to prevent replicative senescence [28]. All experiments with fibroblasts alone were carried out on tissue culture plastic substrata coated with 0.1 mg/ml collagen-I solution (Millipore) overnight at 4°C. Unless otherwise indicated, fibroblasts were seeded at 3.3×10^3^ cells/cm^2^ in all mono-culture experiments. In some experiments, fibroblasts were daily exposed to 4 ng/ml recombinant human TGF-β1 (R&D Systems) continuously or intermitently for 4h/day up to 2 weeks. In other experiments, fibroblasts were treated with 0.15-0.3 mM H_2_O_2_ (Sigma) for 2h to induce oxidative stress, washed and maintained for 4 days as reported elsewhere [17]. For SA-βgal experiments, fibroblasts were seeded on 6-well plates and maintained in serum-free fibroblast culture medium for 3 days. In some experiments, CCD-19Lu fibroblasts were treated with 50 mU/mL bleomycin (Sigma) for 10 days, refreshing the bleomycin containing medium on alternating days. For flow cytometry experiments, fibroblasts were seeded in fibroblast culture medium for 24h and maintained either in 0% FBS (serum-free) or 10% FBS medium for 5 days with a media change at day 3. For immunofluorescence measurements, fibroblasts were seeded at 8×10^3^ cells/cm^2^ on 8-well chamber slides (BD) in serum-free fibroblast culture medium for 3 days.

Co-culture experiments were carried out with a panel of lung cancer cell lines (ATCC, Manassas) originally derived from either ADC (A549, H522), SCC (H520, SK-MES-1) and LCC (H460, H661, H1299) patients (ATCC), and were used up to passage 10. Their p53 status is shown in Supplementary Table S2. Cancer cells were maintained in RPMI-1640 medium supplemented with 10% of FBS (Gibco), 50 IU/mL penicillin, 50 μg/mL streptomycin, 2.5 μg/mL amphotericin B (Sigma), 0.01 M Hepes and 1 mM L-glutamine (Gibco) (referred to as epithelial culture medium). For some experiments, H460 cells were transfected with a miR-200c mimic to render them more epithelial-like [40] and responsive to microenvironmental signals as recently reported by some of us [26]. For this purpose, H460 cells were transiently transfected with 50 nmol/L of the miR-200c mimic pre-miR-200c (Ambion) with Lipofectamine 2000 (Invitrogen), and are referred to as H460R thereafter.

### Heterotypic co-culture of cancer cells and fibroblasts

Indirect co-culture experiments were conducted in Transwell culture plates with inserts (6 Transwell plate, 0.4 μm pore size; Corning). Fibroblasts from non-malignant tissue (either CCD-19Lu or primary CFs) were seeded in a collagen-coated lower Transwell compartment at 5.2×10^3^ cells/cm^2^ in serum-free fibroblast culture medium. Cancer cells were seeded on the Transwell insert at 2.2×10^4^ cells/cm^2^ in serum-free epithelial culture medium at equal volume than fibroblasts for 9-10 days. Bare (cell-free) inserts were used as negative controls. Culture medium was replaced twice weekly. In some co-culture experiments, cells were either daily treated with the antioxidant n-acetyl cysteine (NAC, Sigma) or vehicle or every 2-3 days with the inhibitor of the TGF-β pathway SB505124 (5 μM, Sigma) [41]. For flow cytometry experiments, replicate co-cultures were maintained with 20% FBS epithelial culture medium to render a final 10% FBS concentration upon mixing with fibroblast medium.

### Conditioned medium (CM)

Fibroblasts were co-cultured in Transwells with either bare or cancer cell seeded inserts as described above, which were referred to thereafter as cancer-naive or cancer-educated fibroblasts, respectively. After 9 days, inserts were removed, and fibroblasts were maintained for 4 additional days in serum-free fibroblast culture medium. Afterwards, the fibroblast condition medium (CM) was collected, centrifuged to remove suspended cells, and stored at −20°C until use.

### Immunofluorescence

Immunofluorescence detection and quantification of α-SMA in cultured fibroblasts were carried out as described elsewhere [18, 19].

### Senescence-associated beta-galactosidase activity (SA-βgal)

SA-βgal activity at pH 6.0 was detected following a slightly modified protocol [20]. In brief, fibroblasts were fixed with 4% PFA (Sigma) for 5 min at RT and incubated with SA-βgal staining solution containing 0.2 M citric acid/sodium phosphate buffer (pH 6.0), 100 mM potassium ferricyanide, 100 mM potassium ferrocyanide, 5 M sodium chloride, 2 M magnesium chloride, and 20 mg/mL X-gal (5-Bromo-4-chloro-3-indolyl β-D-galactopyranoside) (Sigma) at 37°C in a stove for ~12h. For each experimental condition, 9 randomized images were obtained with a phase-contrast microscope (Nikon) provided with a F-145C2 Marlin color camera (Allied Vision Technologies) using Metamorph software (Molecular Devices) and a ×20 objective. The percentage of SA-βgal+ (blue) cells was assessed for each image, and averaged for each experimental condition. In average, ~800 fibroblasts were counted per condition.

### Flow cytometry

Cell cycle analysis was performed from DNA histograms of single cells obtained by flow cytometry (LSRFortessa, BD Biosciences) as reported elsewhere [18]. The percentages of arrested fibroblasts (i.e. cells in G0/G1) were assessed with FACSDiva Software v6.1.3 (BD).

### Cell spreading

Phase contrast images acquired to assess SA-β-gal were also used to assess cell spreading by manually outlining the contour of individual cells with Image J, and averaging the corresponding spreading areas for each patient.

### Cell density

Cancer cells were plated at 1.1×10^3^ cells/cm^2^ in 6 well plates and grown in 2 ml of a 1:1 mixture of CM and serum-free epithelial culture medium for 3 days. A 1:1 mixture of serum-free fibroblast/epithelial culture medium was used as (basal) control medium. Cell density was computed as described [18]. In brief, cells were fixed with 4% PFA and their nuclei counterstained with Hoechst 33342 (Molecular Probes). Fluorescent nuclei were imaged and counted with Image J. Cell density was assessed as the average nuclear density/image, and normalized to the value obtained with control medium.

### Invasion

Cancer cell invasion was assessed with a slightly modified Transwell-based assay [37], and performed with Transwell culture plates with filter-inserts (24 Transwell plate, 8 μm filter pore size; Merck Millipore). Matrigel (BD) was diluted in serum-free epithelial culture medium (1:5). Filters were coated with 50 μl diluted Matrigel and incubated for 2h at 37°C. Cancer cells were immediately seeded on the Matrigel-coated filter-inserts as 0.2×10^3^ cells/cm^2^ in serum-free epithelial culture medium. CM or control medium (see cell density) was added to the lower Transwell compartment. After 24h, cells were fixed with 100% methanol for 15 min, and incubated with 0.5% crystal violet (Sigma) for 30 min at RT. Cells on the apical side of the filter-inserts were scraped off. Cells that had invaded into the lower side of the filter were visualized by a phase-contrast microscope provided with a color camera and a ×10 objective. Images of 9 random fields were obtained per condition. Crystal violet positive area per image was assessed using color thresholding with Image J. Invasion was computed as the average crystal violet positive area/image, and normalized to that obtained in control medium.

### *In vivo* tumorigenecity assay

Tumorigenic assays were carried out using CD1-nude female mice (Charles River Laboratories) 5-10 weeks old, which were maintained and manipulated using protocols approved by the Ethics Committee of the *Fondazione IRCCS INT* according to EU Directive 2010/63/EU. H460 or H460R cells (1×10^3^) suspended in 0.1 mL Matrigel (BD Biosciences) were subcutaneously injected into both flanks alone or with CCD-19Lu fibroblasts (3×10^3^) that exhibited either a normal or a senescent phenotype (n = 8/cell condition). Senescence was induced by co-culture with H460 cells as described above. Tumor growth was monitored for 40 days by assessing tumor volume as 0.5×width^2^×length [35]. At the end of the observation period or when tumors reached at least 300 mm^3^, lungs were removed and dissociated for further analysis.

### *In vivo* tumor dissemination

To assess tumor dissemination in tumor-bearing mice, lungs from randomly selected animals (n = 4/cell condition) were removed at either 40 or 50 days after injection as described elsewhere [39], finely minced using a scalpel blade, enzymatically digested for 1h at 37°C with Tumor Dissociation Kit in Gentle Macs Octo Dissociator (Miltenyi Biotec) and filtered through a 100 μm cell strainer (BD falcon). Red blood cells were removed by Lysing Buffer 1X (Becton Dickinson). Disseminated human tumor cells were identified by flow cytometry using a negative gating strategy to exclude both 7-aminoactinomycin D (7-AAD+ non viable) cells and mouse H2K+ cells as recently reported by some of us [39]. In brief, lung cell suspensions were washed and incubated in staining solution containing 1% BSA, 2 mM EDTA, PerCP-eFluor 710 anti-mouse MHC class I (dilution 1:50, e-Bioscence) and 7-AAD viability staining solution (dilution 1:10, e-Bioscence). Fluorescence was detected with a flow cytometer in the far red range using a 650 nm long-pass filter. Final amount of lung disseminated tumor cells was expressed as percentage of 7-AAD and mouse H2K negative cells. This approach was able to specifically detect as few as 10^3^ single human cells in murine lungs [39].

### Statistical analysis

Two group comparisons were performed with Student's *t*-test (SigmaPlot) unless otherwise indicated. Statistical significance was assumed at *P*<0.05. All data shown are mean ± SD unless otherwise specified.

## SUPPLEMENTARY MATERIAL AND METHODS


