# Supplementary material for: Heterotypic paracrine signaling drives fibroblast senescence and tumor progression of large cell carcinoma of the lung
Source: Oncotarget. 2016 Jun 30;7(50):82324–37. doi: 10.18632/oncotarget.10327 (PMC5347694; doi:10.18632/oncotarget.10327)
Supplement: Supplementary file 1 [file oncotarget-07-82324-s001.pdf]

Heterotypic paracrine signaling drives fibroblast senescence and tumor progression of large cell carcinoma of the lung

SUPPLEMENTARY MATERIAL AND METHODS

Lung tissue samples and primary human lung fibroblasts

The mesenchymal nature of the fibroblasts from the collection of the *Universitat de Barcelona/Hospital Clínic* (Barcelona, Spain) was confirmed by their positive and negative immunofluorescence staining with mesenchymal (vimentin) and epithelial (pan-cytokeratin) antibodies, respectively. To confirm the stromal origin of the fibroblasts from the the collection of the *Fondazione IRCCS INT* (Milano, Italy), cultures were immunophenotyped and their mesenchymal nature was confirmed by the positive expression of surface markers CD90, CD166, CD105 and CD73 and negativity for epithelial and hematopoietic markers (EpCAM and CD45).

Isolation of primary mouse lung fibroblasts

Fibroblasts were obtained as tissue explants. For this purpose, skin biopsies were removed from CD1-nude female mice and placed in PBS containing 1% antibiotics/antimycotic solution (penicillin/streptomycin, Gibco-Invitrogen, Carlsbad, CA, USA). Samples were minced and cultured in a solution of 1:1 of RPMI and fibroblast basal medium (PCS-201-041, ATCC) containing 20% FBS and antibiotics. Confluent mouse skin fibroblasts cultures were obtained within 2-3 weeks.

SUPPLEMENTARY FIGURES AND TABLES

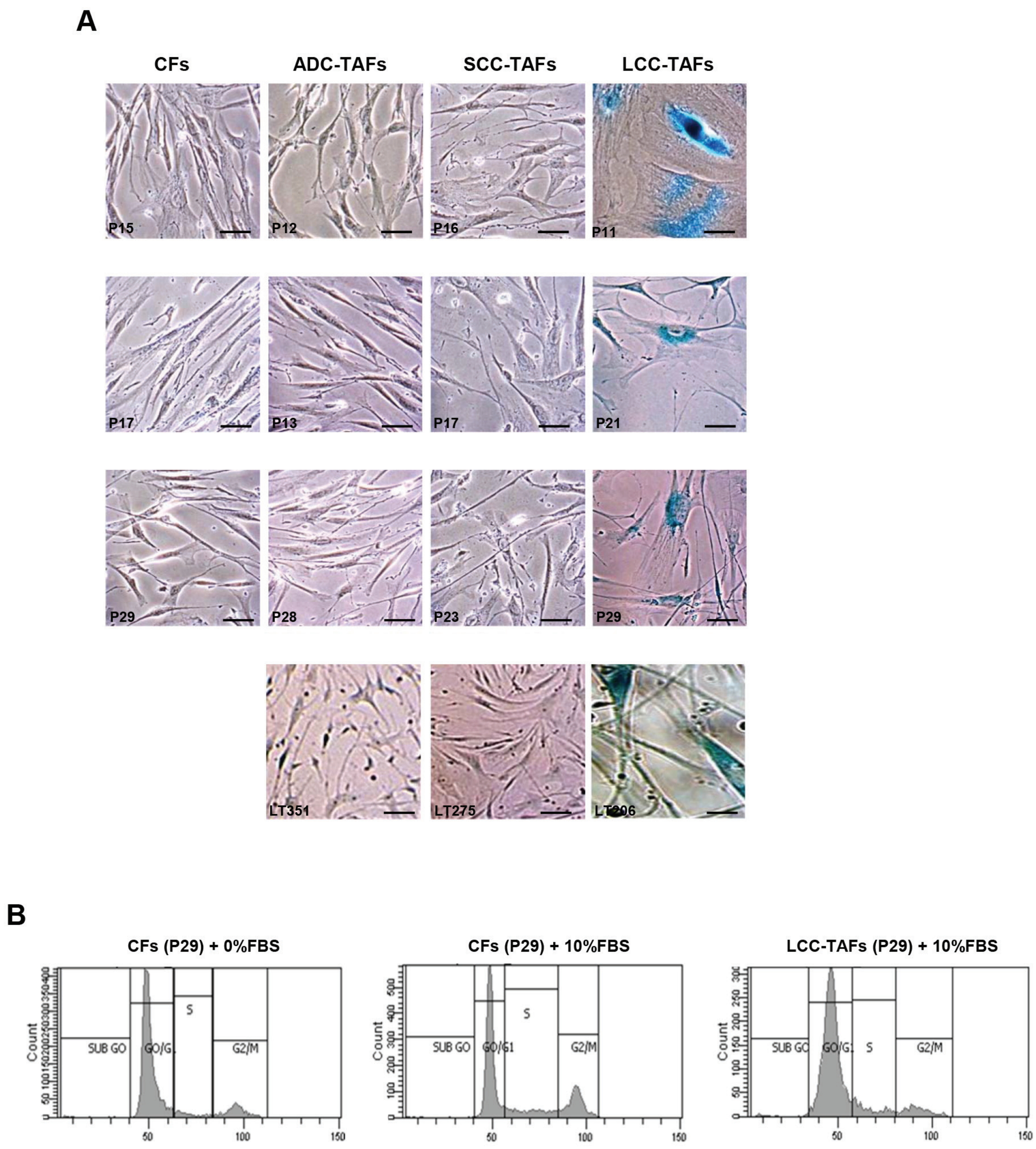

Supplementary Figure S1: A. Representative phase contrast images of SA-βgal stainings of cultured CFs and TAFs from randomly selected patients of each histologic subtype from two independent collections (rows 1-3 are from the *Hospital Clínic* and *Universitat de Barcelona* collection; row are from *Fondazione IRCCS Istituto Nazionale dei Tumori* collection). SA-βgal+ cells appear in blue. Patient number is shown in the bottom-left in each image. Scale bar, 50 µm. **B.** Representative DNA histograms obtained by flow cytometry on CFs and TAFs from a randomly selected LCC patient (P29) cultured in 0% and 10% FBS. Note the absence of the second peak indicative of mitotic cells in TAFs compared to paired CFs upon culture with 10% FBS.

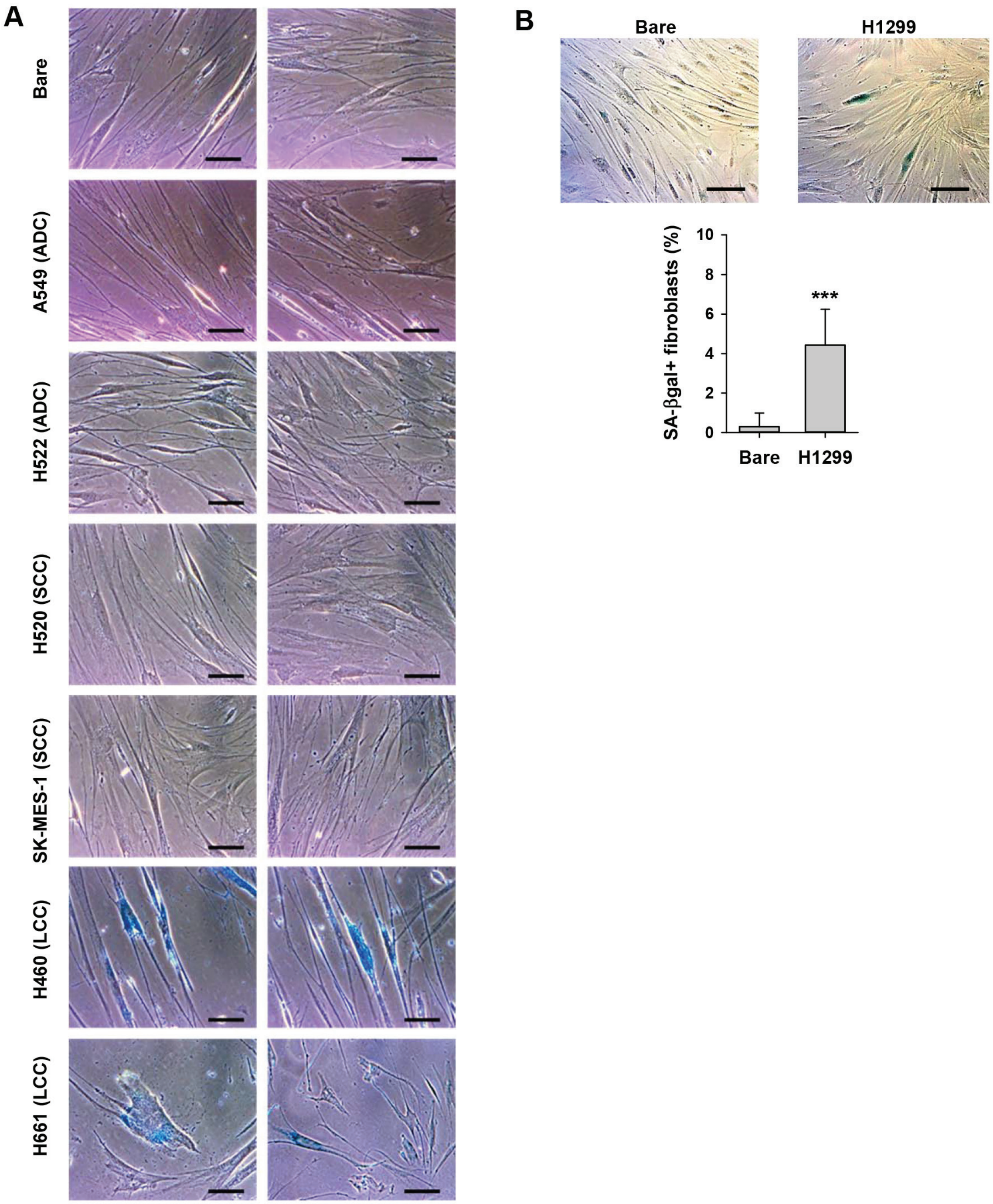

Supplementary Figure S2: A. Representative phase contrast images of SA-βgal stainings of CCD-19Lu fibroblasts co-cultured in Transwells with a panel of lung cancer cell lines derived from ADC (A549, H522), SCC (H520, SK-MES-1) or LCC (H460, H661) patients. Bare Transwell inserts were used as negative control. SA-βgal+ cells appear in blue. Scale bar, 50 µm. **B.** Representative phase contrast images of SA-βgal stainings of CCD-19Lu fibroblasts co-cultured alone or with H1299 LCC cells as outlined in Figure 2A (top). Corresponding average percentage of SA-βgal+ CCD-19Lu fibroblasts are shown below. *, $P < 0.05$; **, $P < 0.01$; ***, $P < 0.005$ (n=2 independent experiments).

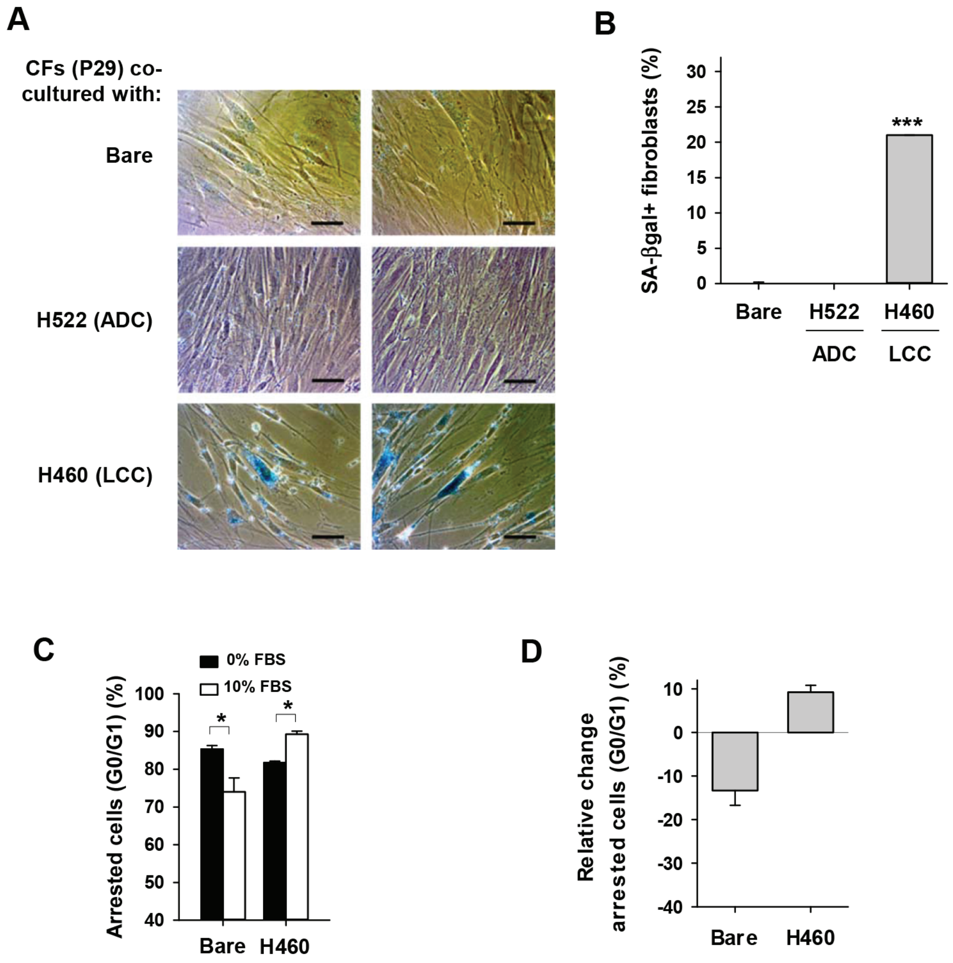

Supplementary Figure S3: A. Representative phase contrast images of SA-βgal stainings of primary control fibroblasts (CFs) isolated from a randomly selected LCC patient (P29) co-cultured with ADC or SCC cancer cell lines. Scale bar, 50 μm. **B.** Average percentage of SA-βgal+ CFs from P29 co-cultured in the conditions described in (A). Bare Transwell inserts were used as negative control. **C.** Average percentage of growth arrested CFs from P29 co-cultured with Bare transwell insert or H460 LCC cancer cells with 0% or 10% FBS. **D.** Average relative changes in growth arrested cells computed from data in (C). *, P <0.05 ; **, P <0.01; ***, P <0.005 (n=2 independent experiments).

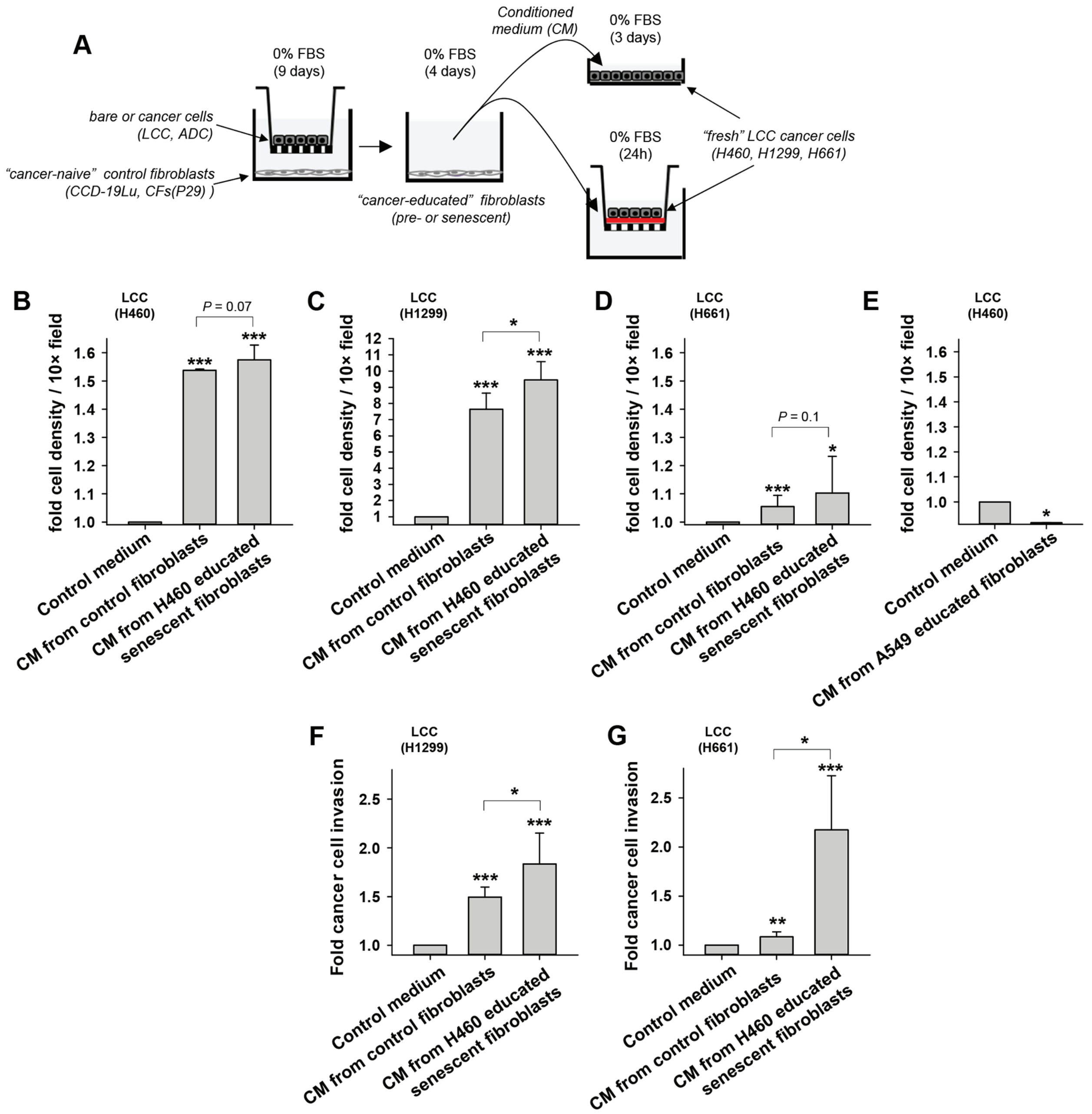

Supplementary Figure S4: A. Outline of the experimental design used to assess growth in cancer cells in culture stimulated with conditioned medium (CM) from fibroblasts co-cultured as in Figure 2A. **B.** Average cell number/field of H460 LCC cells cultured with control medium, CM from cancer-naïve primary CFs from P29, or CM from H460-educated senescent CFs from P29. **C, D.** Average cell number/field of (C) H1299 LCC cells and (D) H661 LCC cells cultured with control medium, CM from cancer-naïve CCD-19Lu fibroblasts, or CM from H460-educated senescent CCD-19Lu fibroblasts. **E.** Average cell number/field of H460 LCC cells cultured with control medium or CM from A549-educated CCD-19Lu fibroblasts. Results were normalized to control medium, and are shown as mean ± SE. **F, G.** Average invasion of either (F) H1299 or (G) H661 cells induced by different types of conditioned medium as in (C,D), respectively. Results were normalized to control medium. *, *P* <0.05 ; **, *P* <0.01; ***, *P* <0.005 (n=2 independent experiments).

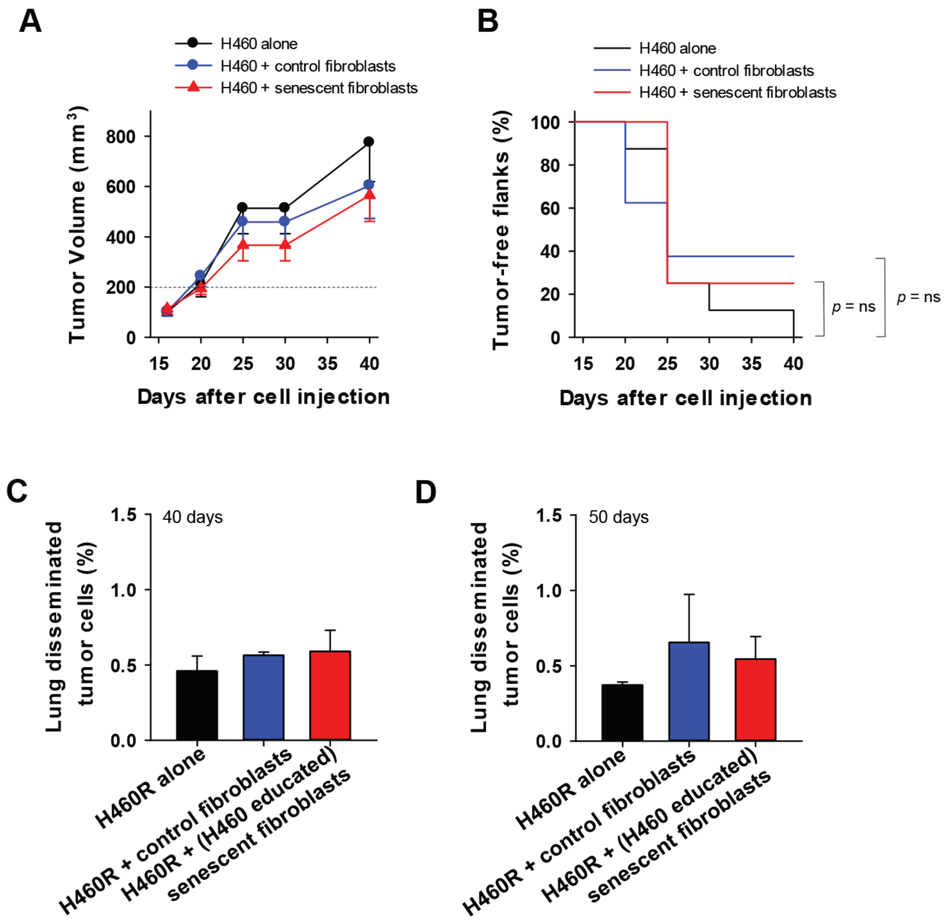

Supplementary Figure S5: A. Tumor volume monitored over 40 days (n = 8 tumors per condition). Data are mean ± SE. **B.** Percentage of tumor-free flanks monitored over 40 days, i.e. tumors lower than 200 mm^3. **C, D.** Percentage of lung disseminated tumor cells at (C) 40 and (D) 50 days after injection. All pair-wise comparisons were performed with respect to H460 injected alone. Statistical significance in (B) was assessed by log-rank test.

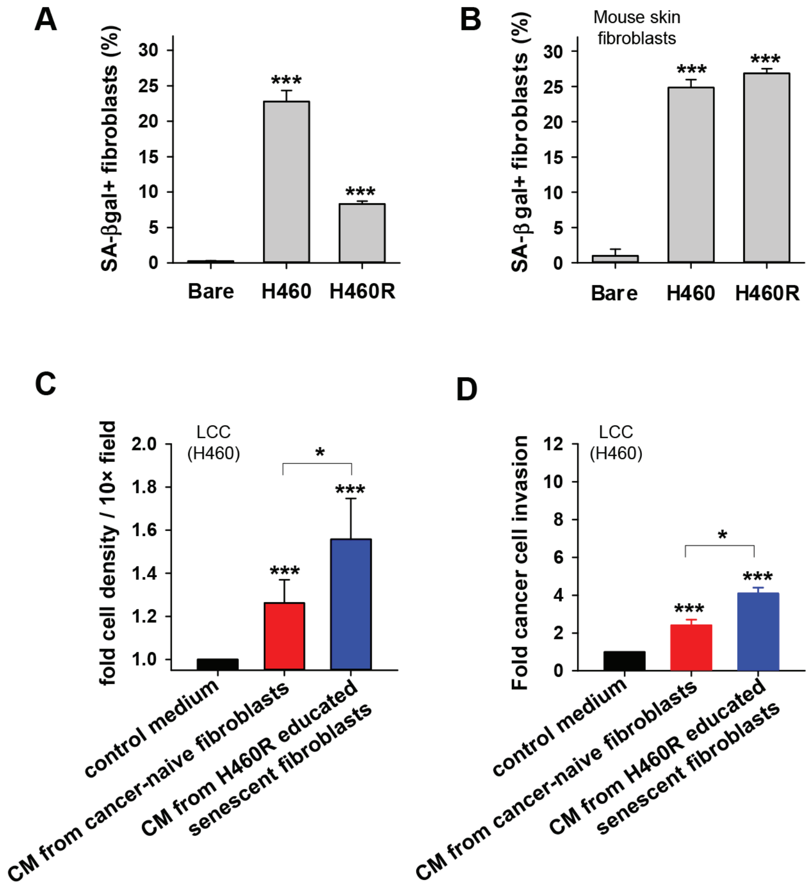

Supplementary Figure S6: A, B. Average percentage of SA-βgal+ CCD-19Lu fibroblasts (A) or primary mouse skin fibroblasts from CD1-nude female mice (B) co-cultured with either parental H460 or H460 cells transfected with a miR-200c mimic (referred to as H460R). Bare Transwell inserts were used as negative control. Details on the isolation of mouse fibroblasts are given in Supplementary Material. **C.** Average cell number/field of H460 LCC cells cultured with control medium or conditioned medium from CCD-19Lu fibroblasts co-cultured either alone, with H460 cells or H460R cells as outlined in Figure 4A. Results were normalized to control medium, and are shown as mean ± SE. **D.** Average invasion of H460 cells induced by different types of conditioned medium as in (C). Results were normalized to control medium. *, P <0.05 ; **, P <0.01; ***, P <0.005 (n=2 independent experiments).

Supplementary Table S1: Summary of patient's clinical data and corresponding percentages of SA-β-gal+ tumor associated fibroblasts (TAFs) and paired control fibroblasts (CFs) in culture from 2 independent collections. Percentages of SA-β-gal+ cells larger than 3% are highlighted in bold

Collection	Patient Ref.	Age (y.o.)	Histologic Subtype	pT	pN	Stage	CFs % SA-β-gal+	TAFs % SA-β-gal+
1. *Hospital Clínic* and *Universitat de Barcelona* (Barcelona, Spain)	P3	72	SCC	T3	N0	IIB	n.a.	n.a.
	P4	73	SCC	T2b	N1*	IIB	n.a.	n.a.
	P5	69	SCC	T2b	N2	IIIA	0.2	0.0
	P6	65	SCC	T1a	N0	IA	0.0	0.0
	P7	61	ADC	T1b	N0	IA	1.8	**18.7**
	P9	75	ADC	T1b	N0	IA	0.0	0.0
	P11	77	LCC	T2b	N1*	IIB	0.0	**67.7**
	P12	70	ADC	T3	N0	IIB	0.3	0.3
	P13	59	ADC	T2b	N2	IIIA	0.4	0.9
	P14	62	SCC	T3	N1	IIIA	0.0	0.0
	P15	73	ADC	T1a	N0	IA	0.0	0.0
	P16	64	SCC	T2a	N1	IIA	0.2	0.0
	P17	80	SCC	T2a	N1	IIA	0.0	0.1
	P21	74	LCC (LCNC)	T1b	N0	IA	0.0	**7.6**
	P23	76	SCC	T2a	Nx	-	1.2	**13.7**
	P26	63	ADC	T2a	N0	IB	0.0	0.0
	P27	71	ADC	T1a	N0	IA	0.0	0.0
	P28	80	ADC	T1a	N0	IA	0.0	0.0
	P29	55	LCC	T3	N0	IIB	0.0	**4.3**
2. *Fondazione IRCCS Istituto Nazionale dei Tumori* (Milano, Italy)	LT154	46	ADC	T2a	N2	IIIA	n.a.	**24.5**
	LT206	75	LCC (LCNC)	T2a	N1	IIA	n.a.	**30.4**
	LT275	66	SCC	T3	N1	IIIA	n.a.	2.0
	LT301	61	SCC	T2a	N0	IIA	n.a.	0.0
	LT351	63	ADC	T2a	N0	IB	n.a.	1.0

N1* : direct hilar ganglionar infiltration by the tumor
Nx: no possibility to perform linphadenectomy
LCNC: large cell neuroendocrine carcinoma
n.a: data not available

Supplementary Table S2: p53 status of lung cancer cell lines used in our study (obtained from the TP53 database of the International Agency for Research on Cancer IARC, http://p53.iarc.fr/CellLines.aspx)

Cell line	p53 status
A549 (ADC)	wild-type (wt)
H522 (ADC)	mutated (missense or small frameshift mutations)
H520 (SCC)	mutated (missense or small frameshift mutations)
SK-MES-1 (SCC)	mutated (missense or small frameshift mutations)
H460 (LCC)	wt
H661 (LCC)	mutated (missense or small frameshift mutations)
H1299 (LCC)	null
